# The genome sequence of an ichneumonid wasp, *Venturia canescens* (Gravenhorst, 1829) (Hymenoptera: Ichneumonidae)

**DOI:** 10.12688/wellcomeopenres.26322.1

**Published:** 2026-04-22

**Authors:** Liam M. Crowley, University of Oxford and Wytham Woods Genome Acquisition Lab, Darwin Tree of Life Barcoding Collective, Wellcome Sanger Institute Tree of Life Management, Samples and Laboratory team, Wellcome Sanger Institute Scientific Operations: Sequencing Operations, Wellcome Sanger Institute Tree of Life Core Informatics team, Tree of Life Core Informatics collective, Darwin Tree of Life Consortium

**Affiliations:** 1 University of Oxford, Oxford, England, UK

**Keywords:** Venturia canescens, Ichneumonid wasp, genome sequence, chromosomal, Hymenoptera

## Abstract

We present a genome assembly from an individual female *Venturia canescens* (ichneumonid wasp; Arthropoda; Insecta; Hymenoptera; Ichneumonidae). The genome sequence has a total length of 299.96 megabases. Most of the assembly (97.25%) is scaffolded into 11 chromosomal pseudomolecules. The mitochondrial genome has also been assembled, with a length of 27.35 kilobases. Gene annotation of this assembly on Ensembl identified 14 281 protein-coding genes. This assembly was generated as part of the Darwin Tree of Life project, which produces reference genomes for eukaryotic species found in Britain and Ireland.

## Species taxonomy

Eukaryota; Opisthokonta; Metazoa; Eumetazoa; Bilateria; Protostomia; Ecdysozoa; Panarthropoda; Arthropoda; Mandibulata; Pancrustacea; Hexapoda; Insecta; Dicondylia; Pterygota; Neoptera; Endopterygota; Hymenoptera; Apocrita; Ichneumonoidea; Ichneumonidae; Campopleginae; Dusona group; *Venturia*; *Venturia canescens* (Gravenhorst, 1829) (NCBI:txid32260).

## Background

*Venturia canescens* (Gravenhorst) (Hymenoptera: Ichneumonidae) is a solitary koinobiont endoparasitoid wasp that develops as a larva within the living larvae of lepidopteran hosts. As a koinobiont, the wasp allows its host to continue developing after oviposition, in contrast to idiobiont parasitoids, which permanently paralyse or kill the host immediately. It is an effective parasitoid of stored-product pest insects, targeting larvae of species such as *Plodia interpunctella* (Indian meal moth) and *Ephestia kuehniella* (Mediterranean flour moth), among other pyralid and phycitid moths ( Mao *et al.*, 2023). The female wasp oviposits directly into host larvae, which continue to develop during parasitism before eventually being killed by the developing wasp larva.

An interesting characteristic of *V. canescens* is its immune evasion strategy. During parasitism, the wasp produces virus-like particles (VcVLPs) that protect eggs against host immune defences ( Mao *et al.*, 2023). These particles are produced in the calyx cells during the wasp’s pupal stage and attach to egg surfaces during maturation ( Mao *et al.*, 2023). Hosts parasitised by *V. canescens* remain capable of mounting an encapsulation response against many foreign targets, but are unable to encapsulate *V. canescens* eggs because of these VLPs on their surface ( Mao *et al.*, 2023). Unlike bracoviruses found in other parasitoid wasps, VcVLPs do not package nucleic acids, instead incorporating wasp-derived virulence proteins hypothesised to cloak the egg from the host’s immune system ( Mao *et al.*, 2023).

The species is also notable for its reproductive polymorphism. *V. canescens* has two reproductive modes — thelytoky (asexual) and arrhenotoky (sexual) — and occurs in habitats with highly variable or relatively stable host abundances, respectively ( Thiel *et al.*, 2006). Thelytokous (asexual) wasps are mostly found in anthropogenic habitats such as grain stores and bakeries, where hosts tend to aggregate and food is absent, while arrhenotokous (sexual) wasps are found in natural habitats such as orchards, where hosts are scattered and food is present ( Pelosse *et al.*, 2007). The species is synovigenic, meaning females continue to mature eggs throughout adult life rather than emerging with a fixed complement, supplementing their energy reserves through feeding on nectar or other sugar sources ( Eliopoulos *et al.*, 2003).

*V. canescens* has a wide distribution, recorded across Europe and beyond, and is cosmopolitan in association with stored-product environments wherever its moth hosts occur. Populations have been collected from flour mills and related facilities, reflecting the wasp’s close association with grain storage and food processing premises ( Eliopoulos *et al.*, 2003).

The species has been used as a model organism in ecological and evolutionary research. Parasitoid wasps in general, and *V. canescens* in particular, have long been considered excellent organisms for studying the evolution of reproductive and life-history strategies ( Harvey *et al.*, 2001). It has primarily been used as a model organism for population dynamics, evolutionary ecology, and immunology ( Eliopoulos *et al.*, 2003). More recently, it has served as a model to study the function and evolution of domesticated endogenous viruses (DEVs), representing one of only three known types of nudivirus-derived DEVs found in parasitoid wasps ( Mao *et al.*, 2023).

**Figure 1.**
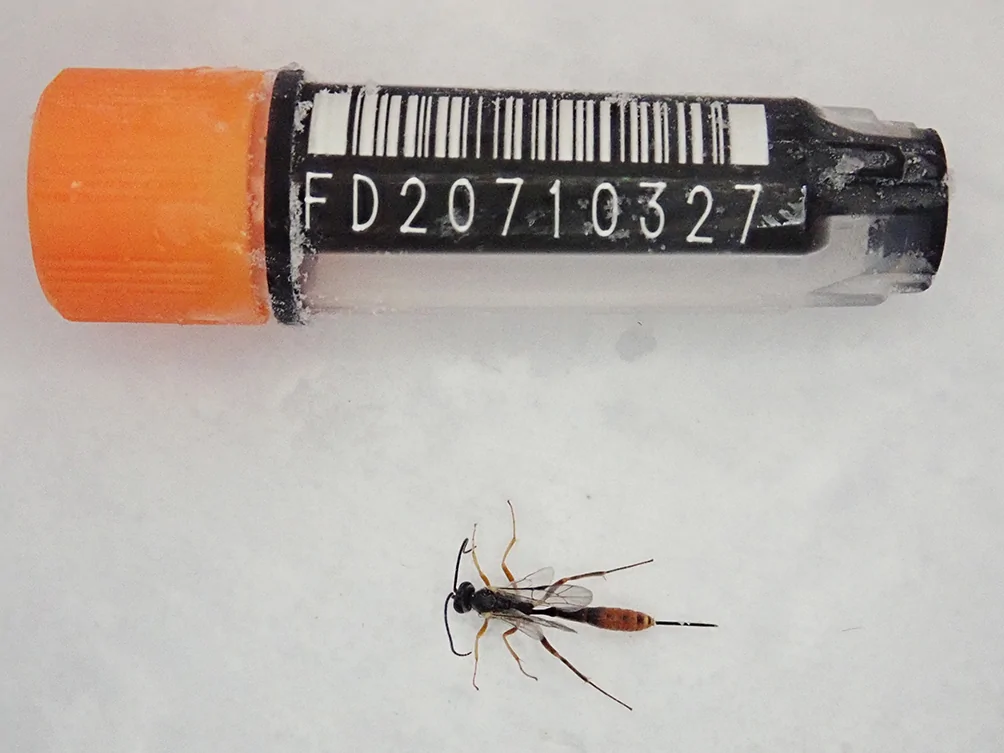
Photograph of the *Venturia canescens* (iyVenCane3) specimen used for genome sequencing.

A recent chromosome-scale genome assembly, comprising 11 chromosomes and 290.8 Mbp, has been produced ( Mao *et al.*, 2023). We present a chromosome-level genome sequence for *Venturia canescens*, generated using the Tree of Life pipeline using a specimen collected from Wytham Woods, Oxfordshire, UK ( Figure 1). This assembly was generated as part of the Darwin Tree of Life Project, which aims to generate high-quality reference genomes for all named eukaryotic species in Britain and Ireland to support research, conservation, and the sustainable use of biodiversity ( Darwin Tree of Life Project Consortium, 2022).

## Methods

### Sample acquisition and DNA barcoding

The specimen used for genome sequencing was an adult female *Venturia canescens* (specimen ID Ox001986, ToLID iyVenCane3; Figure 1), collected from Wytham Woods, Oxfordshire, UK (latitude 51.783, longitude −1.317) on 2022-01-18. Another specimen collected on the same occasion was used for RNA sequencing (specimen ID Ox001987, ToLID iyVenCane4). The specimens were collected and identified by Liam Crowley.

The initial identification was verified by an additional DNA barcoding process according to the framework developed by Twyford *et al.* (2024). A small sample was dissected from the specimen and stored in ethanol, while the remaining parts were shipped on dry ice to the Wellcome Sanger Institute (WSI) (see the protocol). The tissue was lysed, the COI marker region was amplified by PCR, and amplicons were sequenced and compared to the BOLD database, confirming the species identification ( Crowley *et al.*, 2023). Following whole genome sequence generation, the relevant DNA barcode region was also used alongside the initial barcoding data for sample tracking at the WSI ( Twyford *et al.*, 2024). The standard operating procedures for Darwin Tree of Life barcoding are available on protocols.io.

### Nucleic acid extraction

Detailed protocols for nucleic acid extraction developed at the Wellcome Sanger Institute (WSI) Tree of Life Core Laboratory are available on protocols.io ( Howard *et al.*, 2025). The iyVenCane3 sample was weighed and triaged to determine the appropriate extraction protocol. Tissue from the whole organism was homogenised by powermashing using a PowerMasher II tissue disruptor.

High molecular weight (HMW) DNA was extracted in the WSI Scientific Operations core using the Automated MagAttract v2 protocol. DNA was sheared into an average fragment size of 12–20 kb following the Megaruptor®3 for LI PacBio protocol. Sheared DNA was purified by automated SPRI (solid-phase reversible immobilisation). The concentration of the sheared and purified DNA was assessed using a Nanodrop spectrophotometer and Qubit Fluorometer using the Qubit dsDNA High Sensitivity Assay kit. Fragment size distribution was evaluated by running the sample on the FemtoPulse system. For this sample, the final post-shearing DNA had a Qubit concentration of 16.3 ng/μL and a yield of 652.00 ng, with a fragment size of 14.9 kb. The Genomic Quality Number (GQN) was 7.9.

RNA was extracted from whole organism tissue of iyVenCane4 in the Tree of Life Laboratory at the WSI using the RNA Extraction: Automated MagMax™ *mir*Vana protocol. The RNA concentration was assessed using a Nanodrop spectrophotometer and a Qubit Fluorometer using the Qubit RNA Broad-Range Assay kit. Analysis of the integrity of the RNA was done using the Agilent RNA 6000 Pico Kit and Eukaryotic Total RNA assay.

### PacBio HiFi library preparation and sequencing

Library preparation and sequencing were performed at the WSI Scientific Operations core. Libraries were prepared using the SMRTbell Prep Kit 3.0 (Pacific Biosciences, California, USA), following the manufacturer’s instructions. The kit includes reagents for end repair/A-tailing, adapter ligation, post-ligation SMRTbell bead clean-up, and nuclease treatment. Size selection and clean-up were performed using diluted AMPure PB beads (Pacific Biosciences). DNA concentration was quantified using a Qubit Fluorometer v4.0 (ThermoFisher Scientific) and the Qubit 1X dsDNA HS assay kit. Final library fragment size was assessed with the Agilent Femto Pulse Automated Pulsed Field CE Instrument (Agilent Technologies) using the gDNA 55 kb BAC analysis kit.

The sample was sequenced using the Sequel IIe system (Pacific Biosciences, California, USA). The concentration of the library loaded onto the Sequel IIe was in the range 40–135 pM. The SMRT link software, a PacBio web-based end-to-end workflow manager, was used to set-up and monitor the run, and to perform primary and secondary analysis of the data upon completion.

### Hi-C

***Sample preparation and crosslinking***

The Hi-C sample was prepared from 20–50 mg of frozen tissue from the iyVenCane3 sample using the Arima-HiC v2 kit (Arima Genomics). Following the manufacturer’s instructions, tissue was fixed and DNA crosslinked using TC buffer to a final formaldehyde concentration of 2%. The tissue was homogenised using the Diagnocine Power Masher-II. Crosslinked DNA was digested with a restriction enzyme master mix, biotinylated, and ligated. Clean-up was performed with SPRISelect beads before library preparation. DNA concentration was measured with the Qubit Fluorometer (Thermo Fisher Scientific) and Qubit HS Assay Kit. The biotinylation percentage was estimated using the Arima-HiC v2 QC beads.

***Hi-C library preparation and sequencing***

Biotinylated DNA constructs were fragmented using a Covaris E220 sonicator and size selected to 400–600 bp using SPRISelect beads. DNA was enriched with Arima-HiC v2 kit Enrichment beads. End repair, A-tailing, and adapter ligation were carried out with the NEBNext Ultra II DNA Library Prep Kit (New England Biolabs), following a modified protocol where library preparation occurs while DNA remains bound to the Enrichment beads. Library amplification was performed using KAPA HiFi HotStart mix and a custom Unique Dual Index (UDI) barcode set (Integrated DNA Technologies). Depending on sample concentration and biotinylation percentage determined at the crosslinking stage, libraries were amplified with 10–16 PCR cycles. Post-PCR clean-up was performed with SPRISelect beads. Libraries were quantified using the AccuClear Ultra High Sensitivity dsDNA Standards Assay Kit (Biotium) and a FLUOstar Omega plate reader (BMG Labtech).

Prior to sequencing, libraries were normalised to 10 ng/μL. Normalised libraries were quantified again to create equimolar and/or weighted 2.8 nM pools. Pool concentrations were checked using the Agilent 4200 TapeStation (Agilent) with High Sensitivity D500 reagents before sequencing. Sequencing was performed using paired-end 150 bp reads on the Illumina NovaSeq 6000.

### RNA library preparation and sequencing

Libraries were prepared using the NEBNext ^®^ Ultra™ II Directional RNA Library Prep Kit for Illumina (New England Biolabs), following the manufacturer’s instructions. Poly(A) mRNA in the total RNA solution was isolated using oligo (dT) beads, converted to cDNA, and uniquely indexed; 14 PCR cycles were performed. Libraries were size-selected to produce fragments between 100–300 bp. Libraries were quantified, normalised, pooled to a final concentration of 2.8 nM, and diluted to 150 pM for loading. Sequencing was carried out on the Illumina NovaSeq 6000, generating paired-end reads.

### Genome assembly

Prior to assembly of the PacBio HiFi reads, a database of *k*-mer counts ( *k* = 31) was generated from the filtered reads using FastK. GenomeScope2 ( Ranallo-Benavidez *et al.*, 2020) was used to analyse the *k*-mer frequency distributions, providing estimates of genome size, heterozygosity, and repeat content.

The HiFi reads were assembled using Hifiasm ( Cheng *et al.*, 2021) with the --primary option. Haplotypic duplications were identified and removed using purge_dups ( **guanPurgeDups2020?**). The Hi-C reads ( Rao *et al.*, 2014) were mapped to the primary contigs using bwa-mem2 ( Vasimuddin *et al.*, 2019), and the contigs were scaffolded in YaHS ( Zhou *et al.*, 2023) with the --break option for handling potential misassemblies. The scaffolded assemblies were evaluated using Gfastats ( Formenti *et al.*, 2022), BUSCO ( Manni *et al.*, 2021) and MerquryFK ( Rhie *et al.*, 2020).

The mitochondrial genome was assembled using MitoHiFi ( Uliano-Silva *et al.*, 2023).

### Assembly curation

The assembly was decontaminated using the Assembly Screen for Cobionts and Contaminants ( ASCC) pipeline. TreeVal was used to generate the flat files and maps for use in curation. Manual curation was conducted primarily in PretextView and HiGlass ( Kerpedjiev *et al.*, 2018). Scaffolds were visually inspected and corrected as described by Howe *et al*. (2021). Manual corrections included 23 breaks and 218 joins. This reduced the scaffold count by 73.0%, increased the scaffold N50 by 55.0%, and reduced the total assembly length by 9.2%. The curation process is described at https://gitlab.com/wtsi-grit/rapid-curation . PretextSnapshot was used to generate a Hi-C contact map of the final assembly.

### Assembly quality assessment

The MerquryFK tool ( Rhie *et al.*, 2020) was run in a Singularity container ( Kurtzer *et al.*, 2017) to evaluate *k*-mer completeness and assembly quality for the primary and alternate haplotypes using the *k*-mer database ( *k* = 31) computed prior to genome assembly. The analysis outputs included assembly QV scores and completeness statistics.

The genome was analysed using the BlobToolKit pipeline, a Nextflow implementation of the earlier Snakemake version ( Challis *et al.*, 2020). The pipeline aligns PacBio reads using minimap2 ( Li, 2018) and SAMtools ( Danecek *et al.*, 2021) to generate coverage tracks. It runs BUSCO ( Manni *et al.*, 2021) using lineages identified from the NCBI Taxonomy ( Schoch *et al.*, 2020). For the three domain-level lineages, BUSCO genes are aligned to the UniProt Reference Proteomes database ( Bateman *et al.*, 2023) using DIAMOND blastp ( Buchfink *et al.*, 2021). The genome is divided into chunks based on the density of BUSCO genes from the closest taxonomic lineage, and each chunk is aligned to the UniProt Reference Proteomes database with DIAMOND blastx. Sequences without hits are chunked using seqtk and aligned to the NT database with blastn ( Altschul *et al.*, 1990). The BlobToolKit suite consolidates all outputs into a blobdir for visualisation. The BlobToolKit pipeline was developed using nf-core tooling ( Ewels *et al.*, 2020) and MultiQC ( Ewels *et al.*, 2016), with containerisation through Docker ( Merkel, 2014) and Singularity ( Kurtzer *et al.*, 2017).

## Genome sequence report

### Sequence data

**Figure 2.**
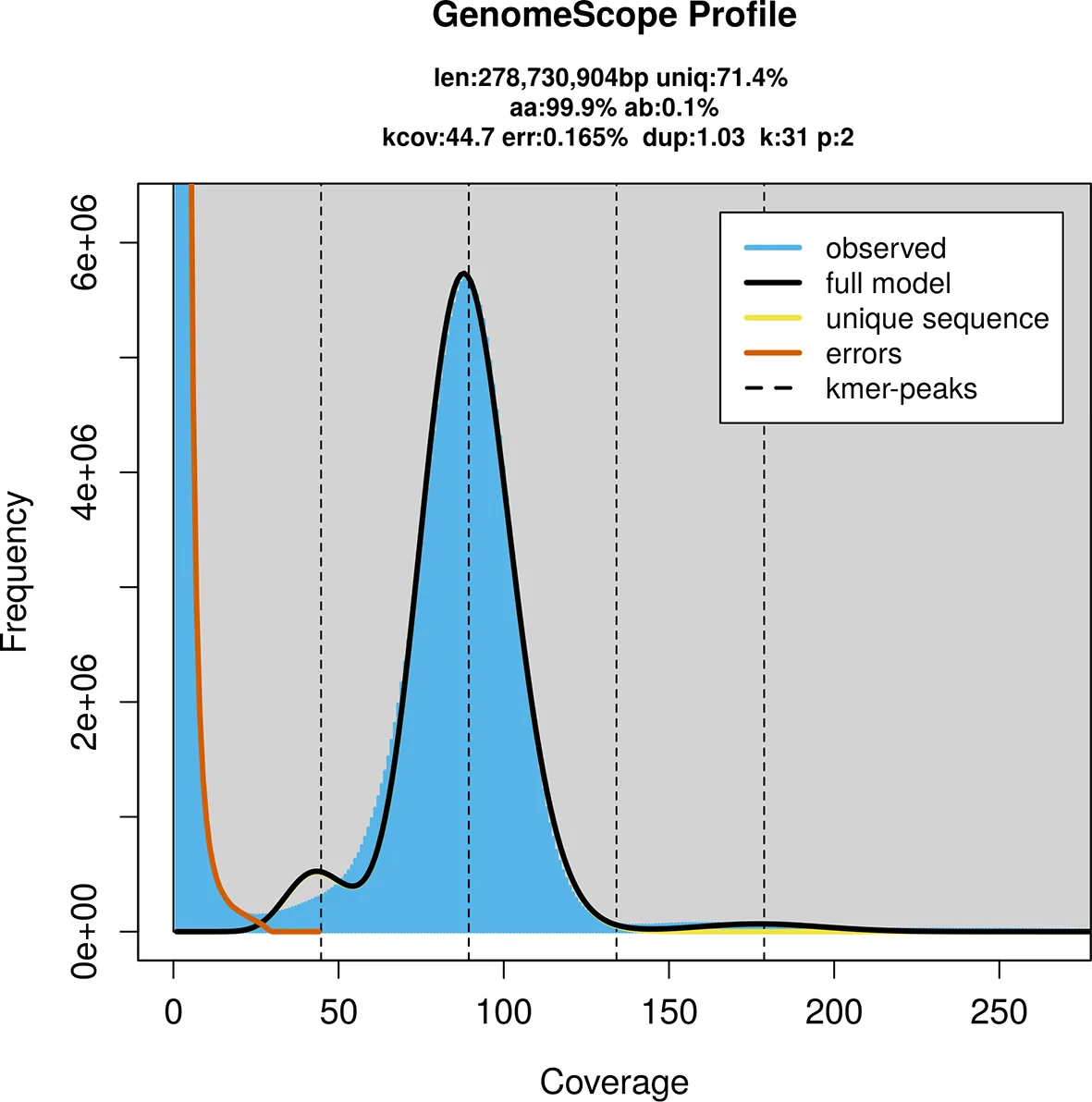
Frequency distribution of *k*-mers generated using GenomeScope2. The plot shows observed and modelled *k*-mer spectra, providing estimates of genome size, heterozygosity, and repeat content based on unassembled sequencing reads.

PacBio sequencing of the *Venturia canescens* specimen generated 26.30 Gb (gigabases) from 2.15 million reads, which were used to assemble the genome. GenomeScope2.0 analysis estimated the haploid genome size at 278.73 Mb, with a heterozygosity of 0.10% and repeat content of 28.70% ( Figure 2). These estimates guided expectations for the assembly. Based on the estimated genome size, the sequencing data provided approximately 89× coverage. Hi-C sequencing produced 107.30 Gb from 355.29 million reads, which were used to scaffold the assembly. RNA sequencing data were also generated and are available in public sequence repositories. Table 1 summarises the specimen and sequencing details.

**Table 1.** Specimen and sequencing data for BioProject PRJEB61493.

Platform	PacBio HiFi	Hi-C	RNA-seq
**ToLID**	iyVenCane3	iyVenCane3	iyVenCane4
**Specimen ID**	Ox001986	Ox001986	Ox001987
**BioSample (source individual)**	SAMEA110451524	SAMEA110451524	SAMEA110451526
**BioSample (tissue)**	SAMEA110451711	SAMEA110451711	SAMEA110451712
**Tissue**	whole organism	whole organism	whole organism
**Instrument**	Sequel	Illumina NovaSeq 6000	Illumina NovaSeq 6000
**Run accessions**	ERR11263497	ERR11271516	ERR12245563
**Read count total**	2.15 million	355.29 million	46.13 million
**Base count total**	26.30 Gb	107.30 Gb	13.93 Gb

### Assembly statistics

The primary haplotype was assembled, and contigs corresponding to an alternate haplotype were also deposited in INSDC databases. The final assembly has a total length of 299.96 Mb in 104 scaffolds, with 321 gaps, and a scaffold N50 of 24.93 Mb ( Table 2).

**Table 2.** Genome assembly data for *Venturia canescens.*

Genome assembly	Primary assembly
**Assembly name**	iyVenCane3.1
**Assembly accession**	GCA_964261885.1
**Alternate haplotype accession**	GCA_964261905.1
**Assembly level**	chromosome
**Span (Mb)**	299.96
**Number of chromosomes**	11
**Number of contigs**	425
**Contig N50**	2.85 Mb
**Number of scaffolds**	104
**Scaffold N50**	24.93 Mb
**Sex chromosomes**	-
**Organelles**	Mitochondrion: 27.35 kb

**Figure 3.**
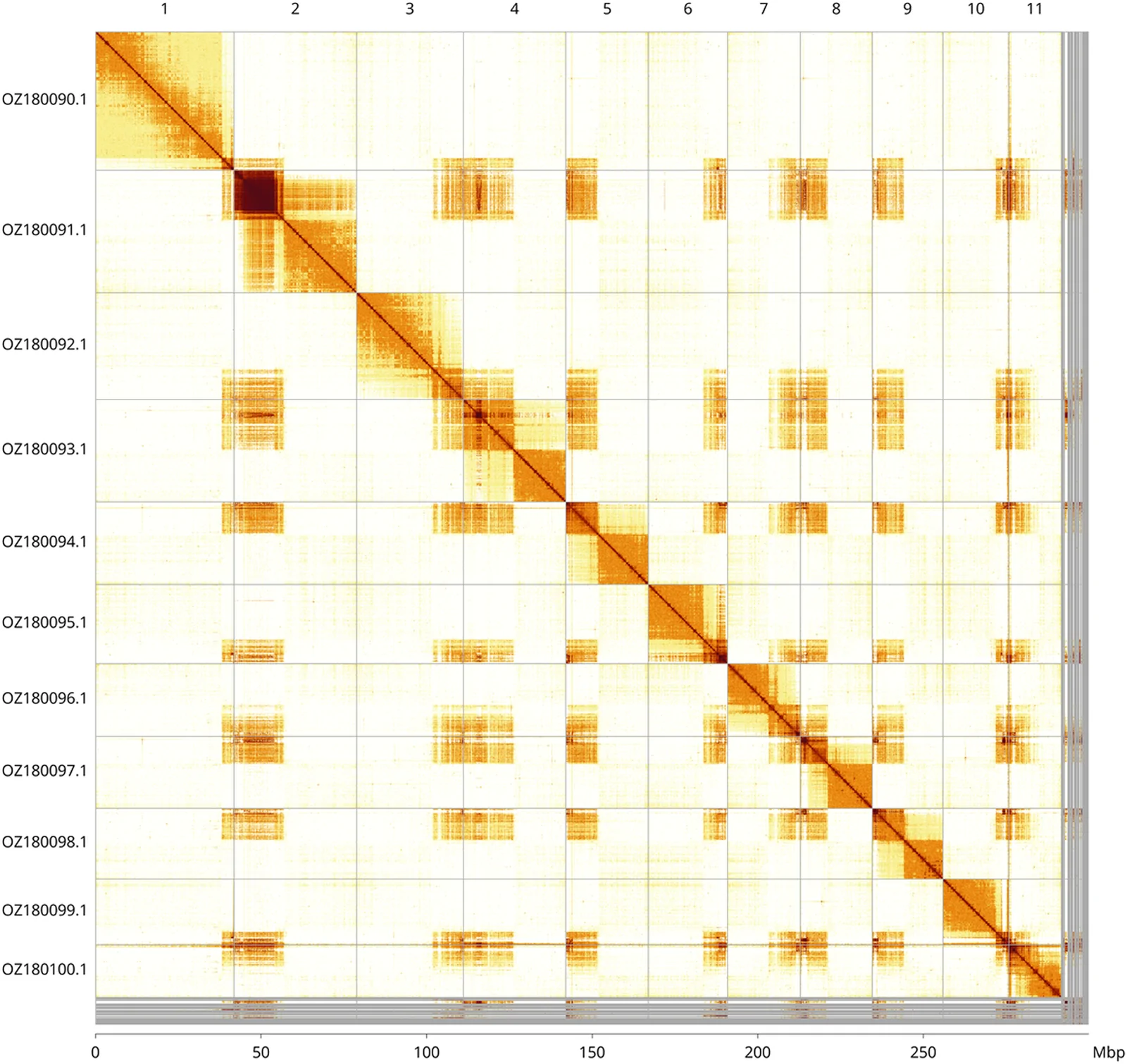
Hi-C contact map of the *Venturia canescens* genome assembly. Assembled chromosomes are shown in order of size and labelled along the axes, with a megabase scale shown below. The plot was generated using PretextSnapshot.

Most of the assembly sequence (97.25%) was assigned to 11 chromosomal-level scaffolds. These chromosome-level scaffolds, confirmed by Hi-C data, are named according to size ( Figure 3; Table 3). The order and orientation of contigs along SUPER_2 between 2.7 Mb and 12.4 Mb is uncertain.

**Table 3.** Chromosomal pseudomolecules in the primary genome assembly of *Venturia canescens* iyVenCane3.

INSDC accession	Molecule	Length (Mb)	GC%
OZ180090.1	1	41.85	38.50
OZ180091.1	2	37	43.50
OZ180092.1	3	32.25	39.50
OZ180093.1	4	30.99	39.50
OZ180094.1	5	24.93	40
OZ180095.1	6	23.86	39.50
OZ180096.1	7	22.02	40
OZ180097.1	8	21.71	40
OZ180098.1	9	21.33	40
OZ180099.1	10	19.91	40
OZ180100.1	11	15.87	39

The mitochondrial genome was also assembled (length 27.35 kb, OZ180101.1). This sequence is included as a contig in the multifasta file of the genome submission and as a standalone record.

### Assembly quality metrics

**Figure 4.**
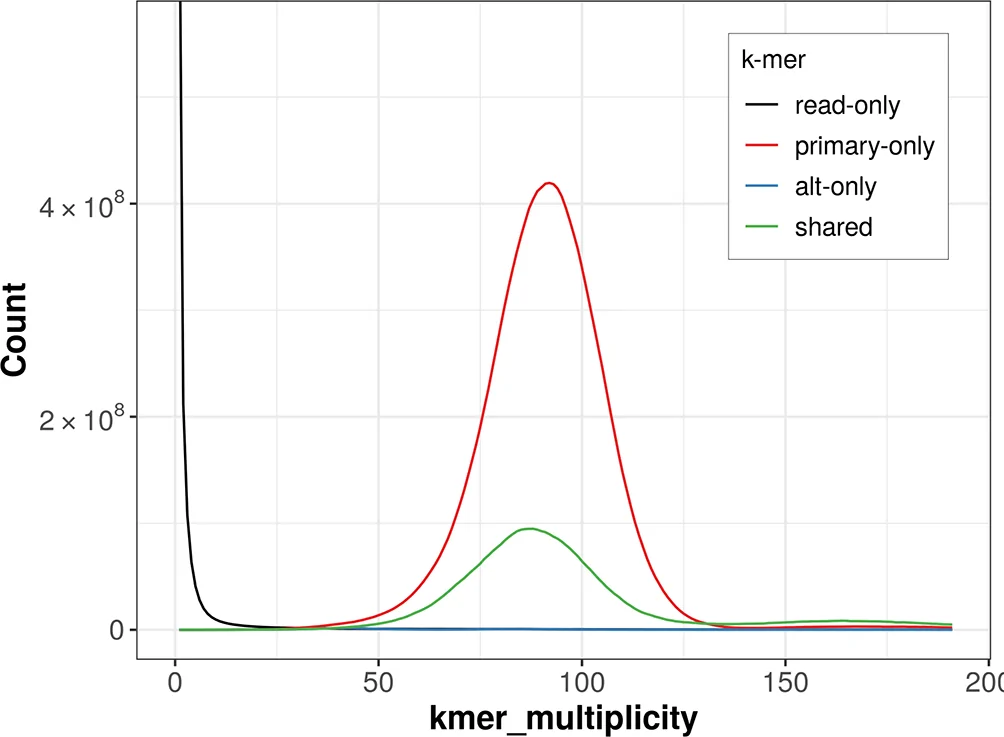
Evaluation of *k*-mer completeness using MerquryFK. This plot illustrates the recovery of *k*-mers from the original read data in the final assemblies. The horizontal axis represents *k*-mer multiplicity, and the vertical axis shows the number of *k*-mers. The black curve represents *k*-mers that appear in the reads but are not assembled. The green curve corresponds to *k*-mers shared by both haplotypes, and the red and blue curves show *k*-mers found only in one of the haplotypes.

The combined primary and alternate assemblies achieve an estimated QV of 59.3. The *k*-mer completeness is 98.69% for the primary assembly, 23.37% for the alternate haplotype, and 99.20% for the combined assemblies ( Figure 4).

**Figure 5.**
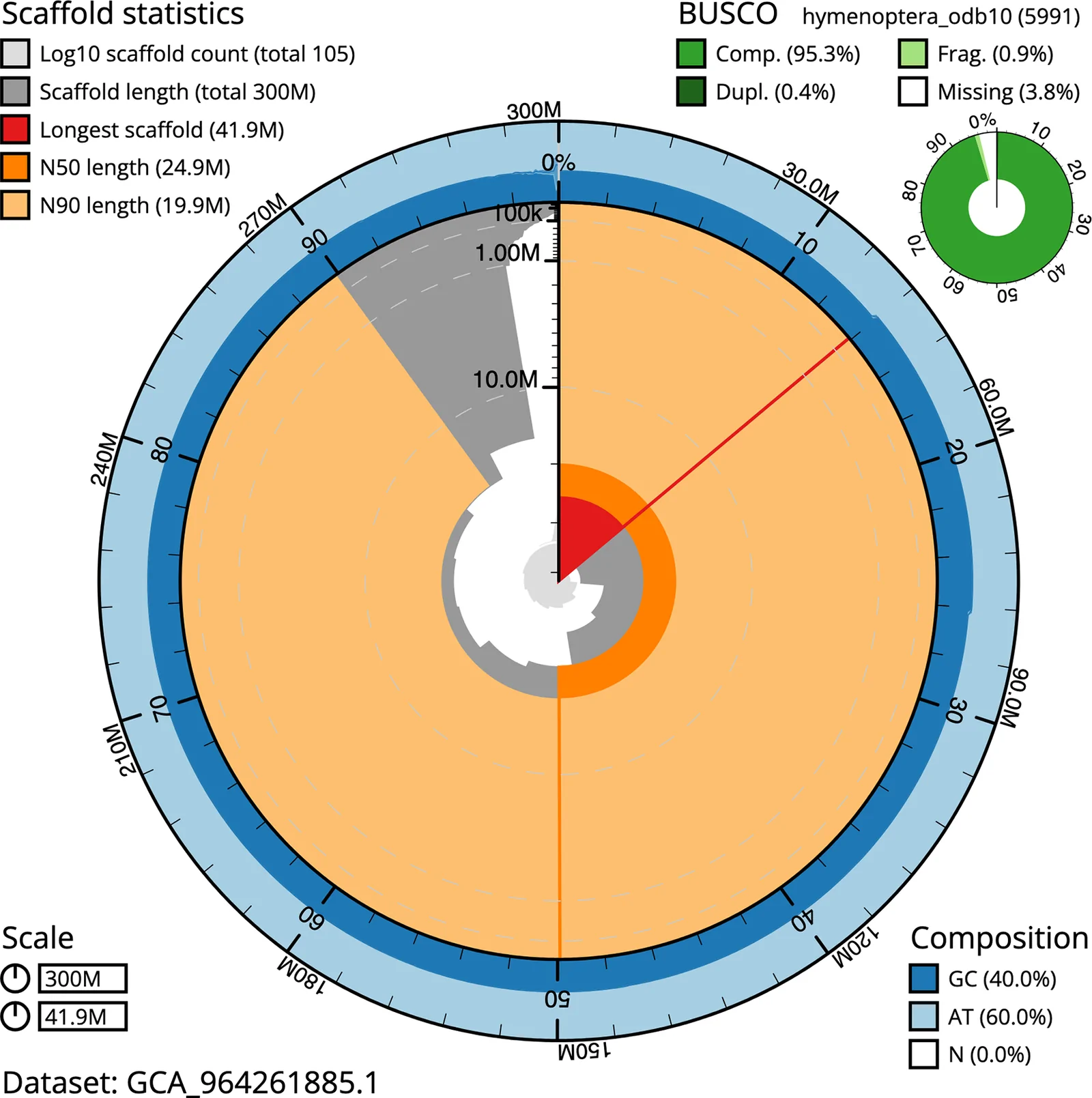
Assembly metrics for iyVenCane3.1. The BlobToolKit snail plot provides an overview of assembly metrics and BUSCO gene completeness. The circumference represents the length of the whole genome sequence, and the main plot is divided into 1 000 bins around the circumference. The outermost blue tracks display the distribution of GC, AT, and N percentages across the bins. Scaffolds are arranged clockwise from longest to shortest and are depicted in dark grey. The longest scaffold is indicated by the red arc, and the deeper orange and pale orange arcs represent the N50 and N90 lengths. A light grey spiral at the centre shows the cumulative scaffold count on a logarithmic scale. A summary of complete, fragmented, duplicated, and missing BUSCO genes in the hymenoptera_odb10 set is presented at the top right. An interactive version of this figure can be accessed on the BlobToolKit viewer.

**Figure 6.**
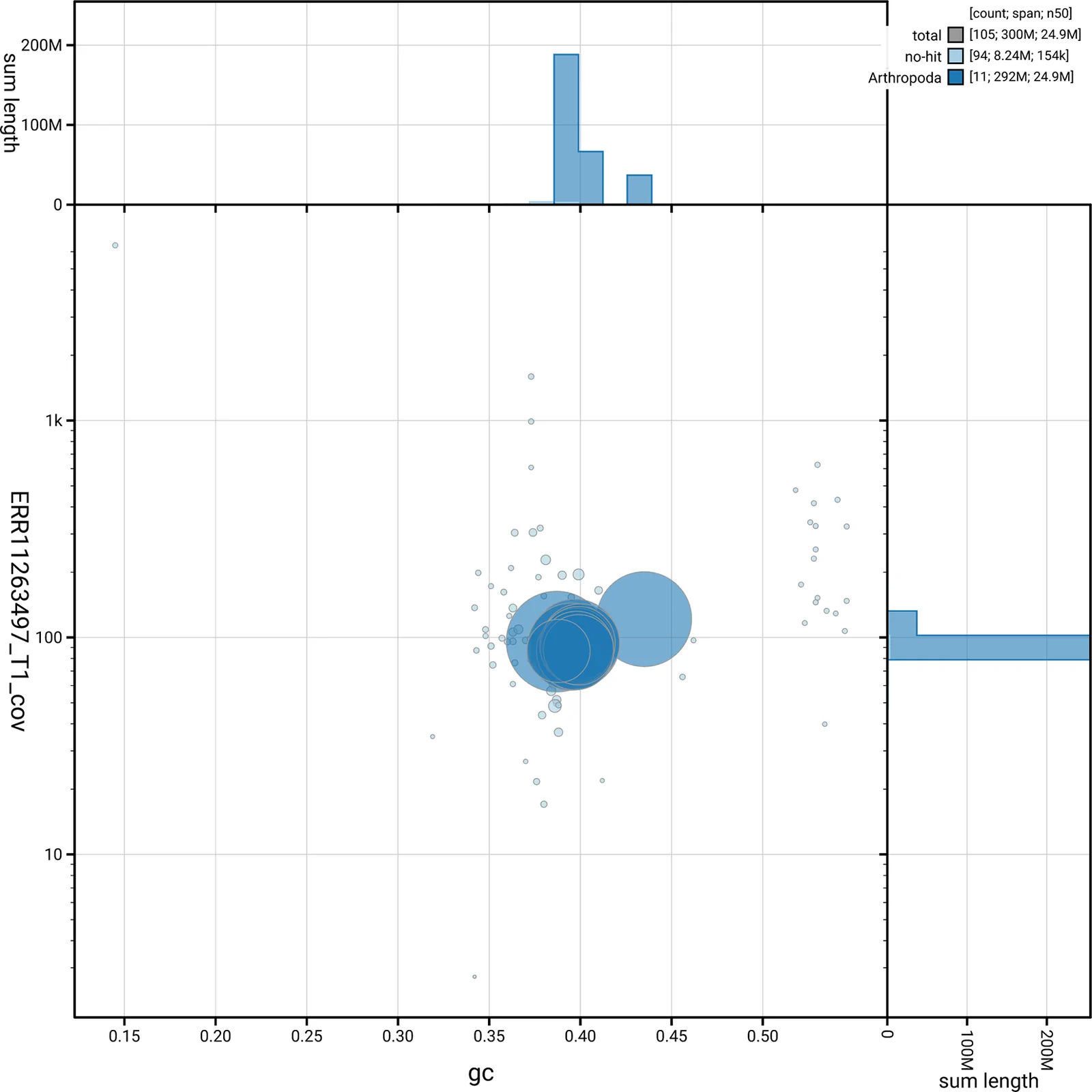
BlobToolKit blob plot for iyVenCane3.1. The plot shows base coverage (vertical axis) and GC content (horizontal axis). The circles represent scaffolds, with the size proportional to scaffold length and the colour representing phylum membership. The histograms along the axes display the total length of sequences distributed across different levels of coverage and GC content. An interactive version of this figure is available on the BlobToolKit viewer.

BUSCO v.5.5.0 analysis using the hymenoptera_odb10 reference set ( *n* = 5 991) identified 95.3% of the expected gene set (single = 94.9%, duplicated = 0.4%). The snail plot in Figure 5 summarises the scaffold length distribution and other assembly statistics for the primary assembly. The blob plot in Figure 6 shows the distribution of scaffolds by GC proportion and coverage.

Table 4 lists the assembly metric benchmarks adapted from Rhie *et al.* (2021) and the Earth BioGenome Project Report on Assembly Standards January 2026. The EBP metric, calculated for the primary assembly, is **6.C.Q64**, meeting the recommended reference standard.

**Table 4.** Earth biogenome project summary metrics for the *Venturia canescens* assembly.

Measure	Value	Benchmark
EBP summary (primary)	6.C.Q64	6.C.Q40
Contig N50 length	2.85 Mb	≥ 1 Mb
Scaffold N50 length	24.93 Mb	= chromosome N50
Consensus quality (QV)	Primary: 64.8; alternate: 55.6; combined: 59.3	≥ 40
*k*-mer completeness	Primary: 98.69%; alternate: 23.37%; combined: 99.20%	≥ 95%
BUSCO	C:95.3% [S:94.9%, D:0.4%], F:0.9%, M:3.8%, n:5 991	S > 90%; D < 5%
Percentage of assembly assigned to chromosomes	97.25%	≥ 90%

**Notes:** The EBP summary uses log10(Contig N50); chromosome-level (C) or log10(Scaffold N50); Q (Merqury QV). BUSCO: C = complete; S = single-copy; D = duplicated; F = fragmented; M = missing; n = orthologues

**Table 5.** Software versions and sources.

Software	Version	Source
BLAST	2.14.0	ftp://ftp.ncbi.nlm.nih.gov/blast/executables/blast+/
BlobToolKit	4.3.9	https://github.com/blobtoolkit/blobtoolkit
BUSCO	5.5.0	https://gitlab.com/ezlab/busco
bwa-mem2	2.2.1	https://github.com/bwa-mem2/bwa-mem2
DIAMOND	2.1.8	https://github.com/bbuchfink/diamond
fasta_windows	0.2.4	https://github.com/tolkit/fasta_windows
FastK	1.1	https://github.com/thegenemyers/FASTK
GenomeScope2.0	2.0.1	https://github.com/tbenavi1/genomescope2.0
Gfastats	1.3.6	https://github.com/vgl-hub/gfastats
Hifiasm	0.16.1	https://github.com/chhylp123/hifiasm
HiGlass	1.13.4	https://github.com/higlass/higlass
MerquryFK	1.1.2	https://github.com/thegenemyers/MERQURY.FK
Minimap2	2.24-r1122	https://github.com/lh3/minimap2
MitoHiFi	2	https://github.com/marcelauliano/MitoHiFi
MultiQC	1.14; 1.17 and 1.18	https://github.com/MultiQC/MultiQC
Nextflow	23.10.0	https://github.com/nextflow-io/nextflow
PretextSnapshot	0.0.5	https://github.com/sanger-tol/PretextSnapshot
PretextView	1.0.3	https://github.com/sanger-tol/PretextView
purge_dups	1.2.3	https://github.com/dfguan/purge_dups
samtools	1.19.2	https://github.com/samtools/samtools
sanger-tol/ascc	0.1.0	https://github.com/sanger-tol/ascc
sanger-tol/blobtoolkit	0.6.0	https://github.com/sanger-tol/blobtoolkit
sanger-tol/curationpretext	1.4.2	https://github.com/sanger-tol/curationpretext
Seqtk	1.3	https://github.com/lh3/seqtk
Singularity	3.9.0	https://github.com/sylabs/singularity
TreeVal	1.4.0	https://github.com/sanger-tol/treeval
YaHS	1.1a.2	https://github.com/c-zhou/yahs

### Genome annotation report

The *Venturia canescens* genome assembly (GCA_964261885.1) was annotated by Ensembl at the European Bioinformatics Institute (EBI). This annotation includes 33 436 transcribed mRNAs from 14 281 protein-coding and 3 443 non-coding genes. The average transcript length is 11 444.03 bp, with an average of 1.89 coding transcripts per gene and 6.02 exons per transcript. Further details of this annotation are available from the Ensembl annotation page.

## Author information

Contributors are listed at the following links:

• Members of the University of Oxford and Wytham Woods Genome Acquisition Lab
• Members of the Darwin Tree of Life Barcoding collective
• Members of the Wellcome Sanger Institute Tree of Life Management, Samples and Laboratory team
• Members of Wellcome Sanger Institute Scientific Operations – Sequencing Operations
• Members of the Wellcome Sanger Institute Tree of Life Core Informatics team
• Members of the Tree of Life Core Informatics collective
• Members of the Darwin Tree of Life Consortium

## Wellcome sanger institute – legal and governance

The materials that have contributed to this genome note have been supplied by a Darwin Tree of Life Partner. The submission of materials by a Darwin Tree of Life Partner is subject to the **‘Darwin Tree of Life Project Sampling Code of Practice’**, which can be found in full on the Darwin Tree of Life website. By agreeing with and signing up to the Sampling Code of Practice, the Darwin Tree of Life Partner agrees they will meet the legal and ethical requirements and standards set out within this document in respect of all samples acquired for, and supplied to, the Darwin Tree of Life Project. Further, the Wellcome Sanger Institute employs a process whereby due diligence is carried out proportionate to the nature of the materials themselves, and the circumstances under which they have been/are to be collected and provided for use. The purpose of this is to address and mitigate any potential legal and/or ethical implications of receipt and use of the materials as part of the research project, and to ensure that in doing so we align with best practice wherever possible. The overarching areas of consideration are:

• Ethical review of provenance and sourcing of the material
• Legality of collection, transfer and use (national and international)

Each transfer of samples is further undertaken according to a Research Collaboration Agreement or Material Transfer Agreement entered into by the Darwin Tree of Life Partner, Genome Research Limited (operating as the Wellcome Sanger Institute), and in some circumstances, other Darwin Tree of Life collaborators.

## Data Availability

European Nucleotide Archive: Venturia canescens. Accession number PRJEB61493. The genome sequence is released openly for reuse. The *Venturia canescens* genome sequencing initiative is part of the Darwin Tree of Life Project (PRJEB40665) and Sanger Institute Tree of Life Programme (PRJEB43745). All raw sequence data and the assembly have been deposited in INSDC databases. Raw data and assembly accession identifiers are reported in Tables 1 and 2. Production code used in genome assembly at the WSI Tree of Life is available at https://github.com/sanger-tol . Table 5 lists software versions used in this study.
