# Supplementary material for: The genome sequence of an ichneumonid wasp, Venturia canescens (Gravenhorst, 1829) (Hymenoptera: Ichneumonidae)
Source: Wellcome Open Res. 2026 Apr 22;11:235. [Version 1] doi: 10.12688/wellcomeopenres.26322.1 (PMC13560598; doi:10.12688/wellcomeopenres.26322.1)
Supplement: Peer review history

# Reviewer report: Reviewer response for version 1

**DOI:** 10.21956/wellcomeopenres.28989.r160822

**Published:** 2026-08-07

**Authors:** Kyle E McElroy

**Affiliations:** 1 Ohio State University, Columbus, Ohio, USA

**Summary**

 The authors describe the sequencing, assembling, and annotation of a genome for the parasitoid wasp *Venturia canescens*. Interesting aspects of the life-history and ecological/economic importance of this species are outlined in the “Background”, including its reproductive polymorphism (i.e., sexual and asexual) and its use of virus-like particles in evading host immune response. The methods for this project and well described, and the resultant chromosome-level genome assembly appears to be of exceptionally high quality and contiguity based on the BUSCO and Merqury completeness scores, as well as the fact that the overwhelming amount of assembled sequence (97.25%) were assigned to one of the 11 chromosomes. This report effectively describes the purpose, methods, and results and I have just a few comments for improvement.

**Minor comments**

 The “Background” section was too reliant on Mao et al. 2023 for general information on the life-history and biology of this species. For example, the entire paragraph about the virus-like particles (second paragraph in “Background”), references Mao et al. four times, despite that paper not being the original source of any of the information. I suggest connecting the relevant primary literature to this paragraph.

 Given that a reference genome was already available for this species (Mao et al. 2023), how does this assembly compare? They seem to be similar in total length (the one described in this report is ~10 Mb larger), does the length difference come from unplaced scaffolds or is there perhaps a repetitive region collapsed in the Mao et al. assembly? Furthermore, with a reference genome already available, what additional information will (or could) be gained from this assembly?

 There was no “Methods” text for the genome annotation. The “Genome annotation report” section in “Genome Sequence Report” states that Ensembl annotated it, but I think at least a brief sketch of what that entails should be included in this report if the results are included.

**Other specific comments**

 “guanPurgeDups2020?” in the “Genome Assembly” section: I think this is a leftover reference placeholder that needs updating.

 “PacBio sequencing of the *Venturia canescens* specimen generated 26.30 Gb (gigabases)” in the “Sequence data” section: the inclusion of “gigabases” here seems unnecessary, or at least inconsistent given that the “Mbp” and “bp” are used throughout the “Background” and “Methods”.

Are sufficient details of methods and materials provided to allow replication by others?

Yes

Is the rationale for creating the dataset(s) clearly described?

Yes

Are the datasets clearly presented in a useable and accessible format?

Yes

Are the protocols appropriate and is the work technically sound?

Yes

Reviewer Expertise:

Comparative genomics, molecular evolution, mollusks, reproductive systems, light-sensing proteins and cells

I confirm that I have read this submission and believe that I have an appropriate level of expertise to confirm that it is of an acceptable scientific standard.

# Reviewer report: Reviewer response for version 1

**DOI:** 10.21956/wellcomeopenres.28989.r158675

**Published:** 2026-07-09

**Authors:** James B Whitfield

**Affiliations:** 1 University of Illinois at Urbana-Champaign, Urbana-Champaign, Illinois, USA

This paper documents the production of a chromosomal-scale annotated genome assembly for the parasitoid wasp Venturia canescens, which is an interesting organism in multiple contexts, including its sex determination variation and its association with endogenous viral elements that play important roles in parasitism of host insects. These contexts are highlighted by the authors in the introduction, who also mention that a chromosomal-scale assembly was already produced by a previous group.

 The methodology and results of this paper all seem appropriate and well described, so that this genome should be available to serve as an important reference for furture studies. It was not clear, however, how this reference genome compares to that produced by the Mao et al. (2023) study that is cited. Presumably it is more complete? Are they highly congruent in content and detail?  A brief mention of how they differ (if at all) would be useful for future users of the genome as a reference.

Are sufficient details of methods and materials provided to allow replication by others?

Yes

Is the rationale for creating the dataset(s) clearly described?

Yes

Are the datasets clearly presented in a useable and accessible format?

Yes

Are the protocols appropriate and is the work technically sound?

Yes

Reviewer Expertise:

Systematics, ecology and viral associations of parasitoid wasps.

I confirm that I have read this submission and believe that I have an appropriate level of expertise to confirm that it is of an acceptable scientific standard.
